# Localizing Epileptic Foci Before Surgery in Patients With MRI-Negative Refractory Epilepsy Using Statistical Parameter Mapping and Three-Dimensional Stereotactic Surface Projection Based on ^18^F-FDG PET

**DOI:** 10.3389/fbioe.2021.810890

**Published:** 2022-01-05

**Authors:** Hailing Zhou, Wei Zhang, Zhiqiang Tan, Ziqing Zhou, Ying Li, Shaojuan Zhang, Lingling Zhang, Jiefeng Gan, Huanhua Wu, Yongjin Tang, Yong Cheng, Xueying Ling, Qiang Guo, Hao Xu

**Affiliations:** ^1^ Department of Nuclear Medicine, PET/CT-MRI Center, Center of Cyclotron and PET Radiopharmaceuticals, The First Affiliated Hospital of Jinan University, Guangzhou, China; ^2^ Epilepsy Center, Guangdong 999 Brain Hospital, Affiliated Brain Hospital of Jinan University, Guangzhou, China

**Keywords:** MRI-negative refractory epilepsy, epileptic foci, fluorine-18-fluorodeoxyglucose positron-emission tomography, statistical parameter mapping, three-dimensional stereotactic surface projection

## Abstract

Patients with refractory epilepsy are not only free of seizures after resecting epileptic foci, but also experience significantly improved quality of life. Fluorine-18-fluorodeoxyglucose positron-emission tomography (^18^F-FDG PET) is a promising avenue for detecting epileptic foci in patients with magnetic resonance imaging (MRI)-negative refractory epilepsy. However, the detection of epileptic foci by visual assessment based on ^18^F-FDG PET is often complicated by a variety of factors in clinical practice. Easy imaging methods based on ^18^F-FDG PET images, such as statistical parameter mapping (SPM) and three-dimensional stereotactic surface projection (3D-SSP), can objectively detect epileptic foci. In this study, the regions of surgical resection of patients with over 1 year follow-up and no seizures were defined as standard epileptic foci. We retrospectively analyzed the sensitivity of visual assessment, SPM and 3D-SSP based on ^18^F-FDG PET to detect epileptic foci in MRI-negative refractory epilepsy patients and obtained the sensitivities of visual assessment, SPM and 3D-SSP are 57, 70 and 60% respectively. Visual assessment combined with SPM or 3D-SSP can improve the sensitivity of detecting epileptic foci. The sensitivity was highest when the three methods were combined, but decreased consistency, in localizing epileptic foci. We conclude that SPM and 3D-SSP can be used as objective methods to detect epileptic foci before surgery in patients with MRI-negative refractory epilepsy. Visual assessment is the preferred method for PET image analysis in MRI-negative refractory epilepsy. When the visual assessment is inconsistent with the patient’s electroclinical information, SPM or 3D-SSP was further selected to assess the epileptic foci. If the combination of the two methods still fails to accurately locate the epileptic foci, comprehensive evaluation can be performed by combining the three methods.

## Introduction

According to results released by the World Health Organization (WHO), approximately 50 million people suffer from epilepsy worldwide (World Health Organization, 2019). Approximately one-third of epilepsy patients, called refractory epilepsy patients, continue to have seizures even after receiving medication (Kwan et al., 2010). Studies have shown that patients with refractory epilepsy are not only free of seizures after surgery, but also experience significantly improved quality of life (Dwivedi et al., 2017; Engel et al., 2012).

At present, there is no unified standard for the location of epileptic foci, and evaluations are performed by a multidisciplinary team. Magnetic resonance imaging (MRI) can be used to identify the direct cause of seizures (Bernasconi et al., 2019). However, MRI results are negative in about 20–30% patients with refractory epilepsy, meaning that they cannot be directly treated with surgery and other methods are needed to assist in locating the epileptic foci (Muhlhofer et al., 2017; von Oertzen et al., 2002). Fluorine-18-fluorodeoxyglucose positron-emission tomography (^18^F-FDG PET) is a promising avenue for detecting epileptic foci (Duncan et al., 2016; Jones and Cascino, 2016). Studies have shown that ^18^F-FDG PET can not only guide a second MRI reading to find hidden lesions, but also provide surgeons with potential epileptic foci areas related to seizures (Chassoux et al., 2010; Rathore et al., 2014). At present, detection of abnormal metabolic areas from ^18^F-FDG PET is mainly performed by visual assessment; however, the detection of epileptic foci by visual assessment is often complicated by a variety of factors in clinical practice.

Several easy imaging methods already exist for objective analysis of ^18^F-FDG PET images. Statistical parameter mapping (SPM) is a voxel-based brain mapping software that has been used in detecting epileptic foci for more than 10 years. Studies have shown that the sensitivity of SPM is higher than that of visual assessment (Archambaud et al., 2013; Kim et al., 2002; Kumar et al., 2010; Wang et al., 2016). However, there are no articles assessing the ability of SPM to localize epileptic foci in patients with MRI-negative refractory epilepsy. Three-dimensional stereotactic surface projection (3D-SSP) is another voxel-based brain mapping software that uses a method similar to SPM. Only one article to date has applied 3D-SSP based on ^18^F-FDG PET data to localize epileptic foci in patients with refractory epilepsy; even so, this study did not include patients with MRI-negative epilepsy, and the localization of epileptic foci was based on preoperative multidisciplinary evaluation rather than postoperative results (Wang et al., 2016). At present, no reports have simultaneously used SPM and 3D-SSP to detect epileptic foci before surgery in patients with MRI-negative refractory epilepsy.

In our study, we used the regions of surgical resection of patients with over 1 year follow-up and no seizures as standard epileptic foci, and then evaluated SPM and 3D-SSP based on ^18^F-FDG PET images to detect epileptic foci before surgery in patients with MRI-negative refractory epilepsy.

## Materials and Methods

### Patients

We retrospectively analyzed 643 patients who received ^18^F-FDG PET examination at the PET CT/MRI center of the First Affiliated Hospital of Jinan University between January 1, 2014 and June 30, 2020 and who were followed-up for more than 1 year after surgical resection. Inclusion criteria: 1) Patients diagnosed with refractory epilepsy according to the standards of the International League Against Epilepsy (ILAE); 2) No structural lesions causing seizures were found at 3.0 T epilepsy-protocol; 3) Age≥5 years; 4) Patients underwent surgical resection and were followed-up for at least 1 year; 5) The postoperative outcomes of patients met Engel’s Class Ⅰ standards of ILAE. Exclusion criteria: 1) 3.0 T MRI epilepsy-protocol could detect structural lesions causing seizures; 2) The postoperative outcomes of patients did not meet Engel’s Class Ⅰ standards of ILAE; 3) Incomplete clinical data of patients; 4) Age<5 years. A total of 91 patients were ultimately included. A total of 91 patients underwent preoperative multidisciplinary evaluation to locate the epileptic foci, 70 of whom underwent stereoelectroencephalography (SEEG) examination before treatment. All 91 patients underwent surgical treatment and were followed-up for more than 1 year.

For SPM analysis, we collected 27 age- and gender-matched controls of relatively normal health, which excluded malignant tumors, lymphomas, and hematological diseases. Subjects underwent full-body ^18^F-FDG PET/CT scans and exhibited no ^18^F-FDG PET abnormalities in the head, as well as no history of neurological disease, psychiatric disease, radiation, chemotherapy, or psychiatric medication.

### MRI Epilepsy-Protocol

MRI was conducted on a 3.0 T MRI scanner (GE Discovery 750, Milwaukee, United States). The MRI epilepsy-protocol used for epilepsy patients at our center consists of an axial three-dimensional brain volume imaging (3D BRAVO) T1-weighted sequence (TR/TE 8.2/3.2, TI 450, matrix 256 × 256, 1.0 mm thickness), a T2-weighted axial sequence (TR/TE 12001/91.1, matrix 512 × 512, 1.0 mm thickness), and a 3D Cube T2 fluid attenuation inversion recovery (FLAIR) coronal sequence (TR/TE 5000/126.4, TI 1615.0, matrix 256 × 256, 1.0 mm thickness). MRI was classified as negative when no structural lesions causing seizures were detected (Bernasconi et al., 2019).

### ^18^F-FDG PET Imaging

Interictal ^18^F-FDG PET scans for all patients were conducted using a GE Discovery PET/CT 690 system (300 mm field of view (FOV), matrix 192 × 192, and 3.3 mm slice thickness). Patients were injected with ^18^F-FDG, and ^18^F-FDG PET images were acquired after a 60 min uptake time. The mean dose of ^18^F-FDG administered was 0.1 mCi/kg body weight (3.7 MBq/kg). The injection of ^18^F-FDG and subsequent ^18^F-FDG PET examination were performed under quiet conditions. Patients were scanned while in a standard awake resting state, closing their eyes and unplugging their ears. All participants fasted for at least 6 h before PET (Hwang et al., 2001).

### Visual Assessment

^18^F-FDG PET images were assessed by two experienced nuclear physicians using the GE AW 4.6 workstation, and we used a colorized scale to detect metabolic changes. The nuclear physicians were blinded to patients’ information, and divided the left and right hemispheres into ten lobes (frontal, parietal, occipital, temporal and insula lobe). Abnormal metabolic regions occurred when one hemisphere was metabolically altered from the opposite hemisphere. The presence of at least one abnormal metabolic region in ^18^F-FDG PET images was considered indicative of potential epileptic foci (Spencer, 1994).

### SPM Analysis

^18^F-FDG PET images were analyzed by SPM12 (Institute of Neurology, University College London) using the Matlab platform (2020a, MathWorks, United StatesA). ^18^F-FDG PET images of epilepsy patients and control subjects were imported into SPM12 in NIFTI format and preprocessed, including the application of spatial standardization and smoothing (FWHM = 8 × 8 × 8 mm^3^). On SPM12, we compared ^18^F-FDG PET images of epilepsy patients to the control group using two independent sample T tests with age and gender as covariates. We set SPM threshold values of *p* < 0.05 (matching K > 0, corrected), *p* < 0.005 (matching K > 200, uncorrected), *p* < 0.001 (matching K > 100, uncorrected), and *p* < 0.0001 (matching K > 50, uncorrected). Areas of abnormal metabolism indicated by SPM were considered to be potential epileptic foci (Mayoral et al., 2016).

### 3D-SSP Analysis

3D-SSP analysis based on ^18^F-FDG PET was conducted using Cortex ID software (University of Washington, Minoshima, United States). The ^18^F-FDG PET images were imported into Cortex ID software in DICOM format, and anatomic standardization was performed through rotational correction and stereotactic realignment. The ^18^F-FDG PET image metabolism data was extracted to the brain surface (3D-SSP images) automatically using the Cortex ID software. The Z value represents the degree of metabolic difference between the patient ^18^F-FDG PET image and healthy controls in the database based on four different reference regions (global, pons, cerebellar, thalamus). Z= (*μ*_normal_ -patients)/δ_normal_. We set Z values of 1.96, 1.64, and 1.28. When the absolute value of a patient result was greater than the set Z values, this was considered as evidence of potential epileptic foci (Minoshima et al., 2020; Mizumura and Kumita, 2006).

### Statistical Analysis

Surgical outcomes were based on the guidelines recommended by ILAE (Wieser et al., 2001). Engel’s Class I: Free of disabling seizures. We considered results of Engel’s Class I as good outcomes. Standard epileptic foci were defined as areas of surgical resection. Abnormal metabolic regions were obtained by the three methods. Correct detection was defined by abnormal metabolic zones is large but including surgical resection site. Correct localization was defined as abnormal metabolic zones is localized and consistent with the surgical resection site. Sensitivity is defined as the ratio of the number of patients correctly detected to the total number of patients. For clinical data before and after surgery, we reported medians (Inter-Quartile Range [IQR]) for continuous variables and percentages (numbers) for categorical variables. The sensitivities of different methods were expressed as percentages. SPM analysis was performed using two independent sample T tests. The sensitivities of different methods were compared using a paired Chi-square test. The above results were analyzed by SPSS26 (IBM SPSS Inc, Chicago, IL, United States), *p* < 0.05 was considered statistically significant.

## Results

### Clinical Data

A total of 91 patients were included, including 64 males and 27 females, with an age range of 5–55 years and a median age of 21 (14–26) years. Other information is shown in Table 1. A total of 27 control subjects were subjected to SPM analysis, including 17 males and 10 females, ranged in age from 10 to 51 years, with a median age of 23 (19–27) years. There were no statistically significant differences in age or sex between the epilepsy and control groups.

**TABLE 1:** Demographics and Clinical Data of participants.

Variable	
Gender	
Male	70% (n = 64)
Female	30% (n = 27)
Age at epilepsy onset, y (IQR)	8 (5–14)
Duration of epilepsy, y (IQR)	10 (4–15)
Age at epilepsy surgery, y (IQR)	21 (14–26)
Auras	37% (n = 34)
Seizure type	
Focal onset	69% (n = 63)
Generalized onset	31% (n = 28)
Seizure frequency	
Daily	34% (n = 31)
Weekly	15% (n = 14)
Monthly	43% (n = 39)
Yearly	8% (n = 7)
Follow up, y (IQR)	2 (2–3)

% (n), percent (number); y, year; IQR, Inter-Quartile Range.

### Sensitivities of Visual Assessment, SPM, 3D-SSP in Detecting Epileptic Foci

**FIGURE 1:**
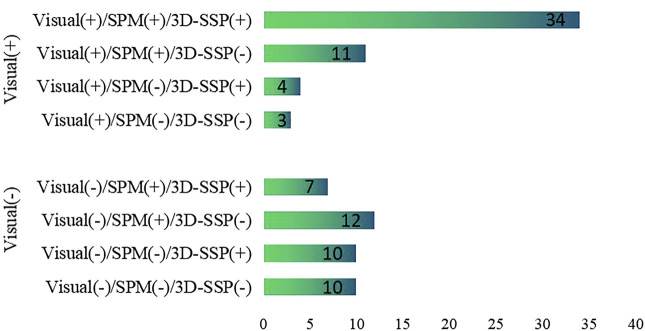
The ability of visual assessment, SPM, 3D-SSP to detect epileptic foci based on postoperative results. Visual, visual assessment; SPM, statistical parameter mapping; 3D-SSP, three-dimensional stereotactic surface projection. (+) = epileptic foci are correctly detected, (−) = epileptic foci are incorrectly detected.

Of 91 patients, epileptic foci were correctly detected for 52 patients, while epileptic foci were incorrectly detected by visual assessment for 39 patients. Among the 52 patients with epileptic foci detected correctly by visual assessment, 45 patients had epileptic foci detected by SPM and 38 patients by 3D-SSP. Among the 39 patients whose visual assessment were incorrect, SPM correctly detected foci in 19 patients, and 3D-SSP correctly detected foci in 17 patients. The abilities of these three methods to detect epileptic foci are shown in Figure 1.

**FIGURE 2:**
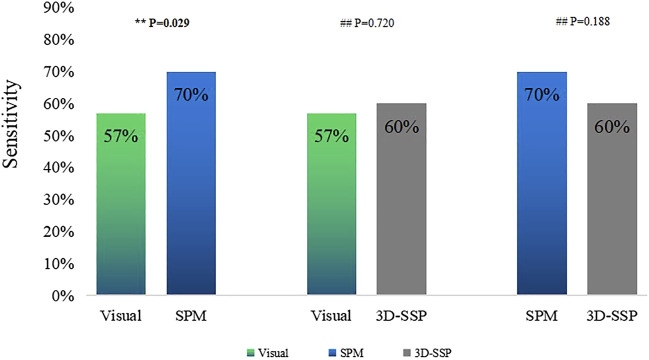
Comparison of the sensitivities of visual assessment, SPM, 3D-SSP in detecting epileptic foci (**P<0.05, ##P>0.05).

Visual assessment, SPM and 3D-SSP were respectively able to detect 52, 64 and 55 patients with respective sensitivities of 57, 70 and 60%. For the three methods respectively, 23, 24 and 27 patients exhibited localized abnormal metabolic regions consistent with surgical resection sites. There was a statistically significant difference between SPM and visual assessment, but there was no statistically significant difference between SPM and 3D-SSP. The sensitivity of visual assessment was similar to that of 3D-SSP, and there was no statistically significant difference detected between them (Figure 2).

### Sensitivity of Two Combined Methods

Visual assessment combined with SPM (Visual/SPM) and visual assessment combined with 3D-SSP (Visual/3D-SSP) were able to detect 71 and 69 patients, respectively, with sensitivities of 78 and 76%. For 8 and 11 patients, respectively, abnormal metabolic regions were localized and consistent with surgical resection sites.

**FIGURE 3:**
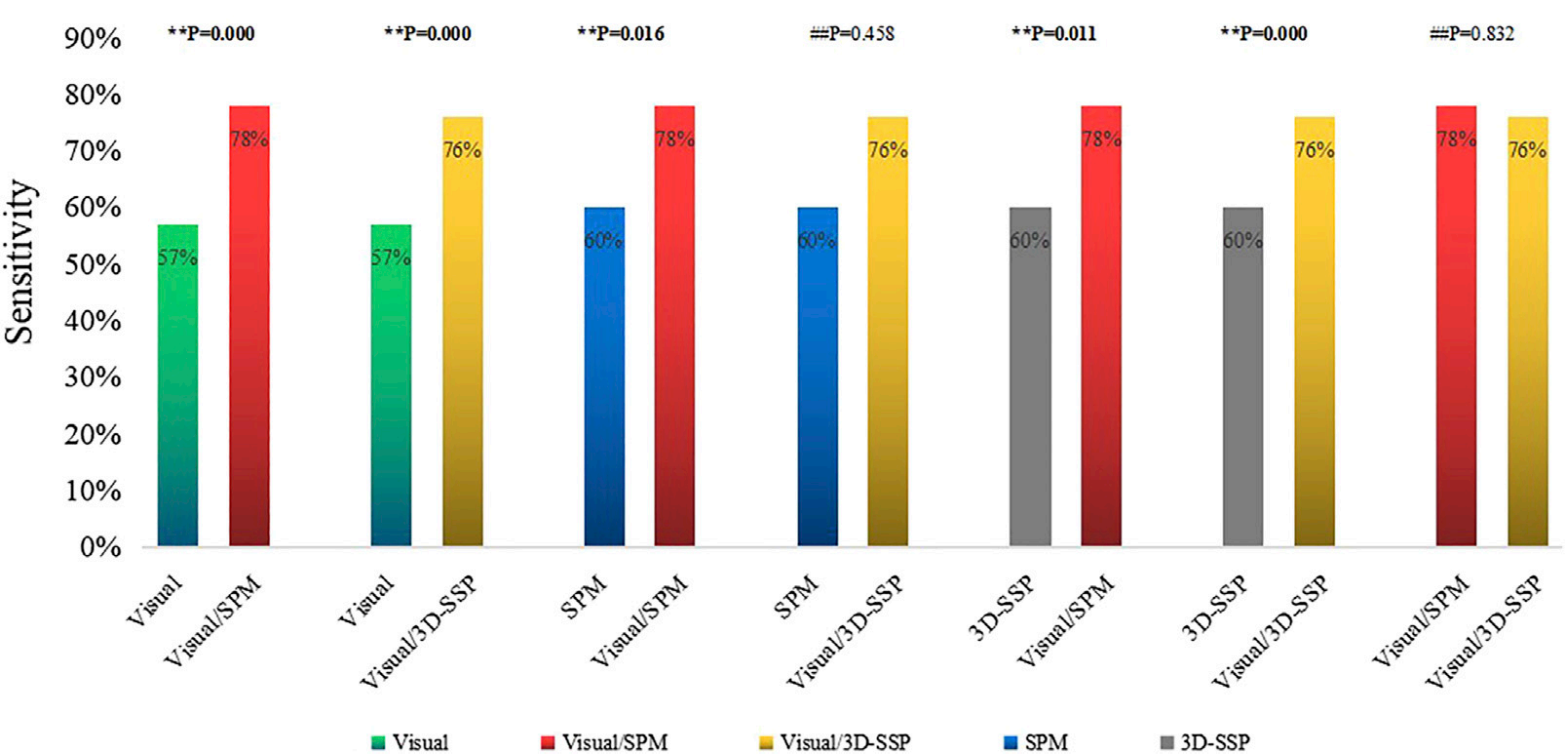
Comparison of the sensitivities of single method, two combined methods (**P<0.05, ##P>0.05). Visual/SPM, Visual assessment combined with SPM; Visual/3D-SSP, Visual assessment combined with 3D-SSP.

The sensitivity of Visual/SPM or Visual/3D-SSP was significantly higher than those of visual assessment alone or of 3D-SSP, and there was a statistically significant difference between them. Although the sensitivities of Visual/SPM and Visual/3D-SSP were higher than that of SPM, there was a statistical difference between Visual/SPM and SPM and no statistical difference between Visual/SPM and 3D-SSP. The sensitivity of Visual/SPM was similar to that of Visual/3D-SSP, and there was no statistically significant difference between the two (Figure 3).

### Sensitivity of Three Combined Methods

Visual assessment combined with SPM and 3D-SSP (Visual/SPM/3D-SSP) could detect epileptic foci in 81 patients with a sensitivity of 89%, and 2 patients had abnormal focal metabolic areas consistent with the surgical resection site.

**FIGURE 4:**
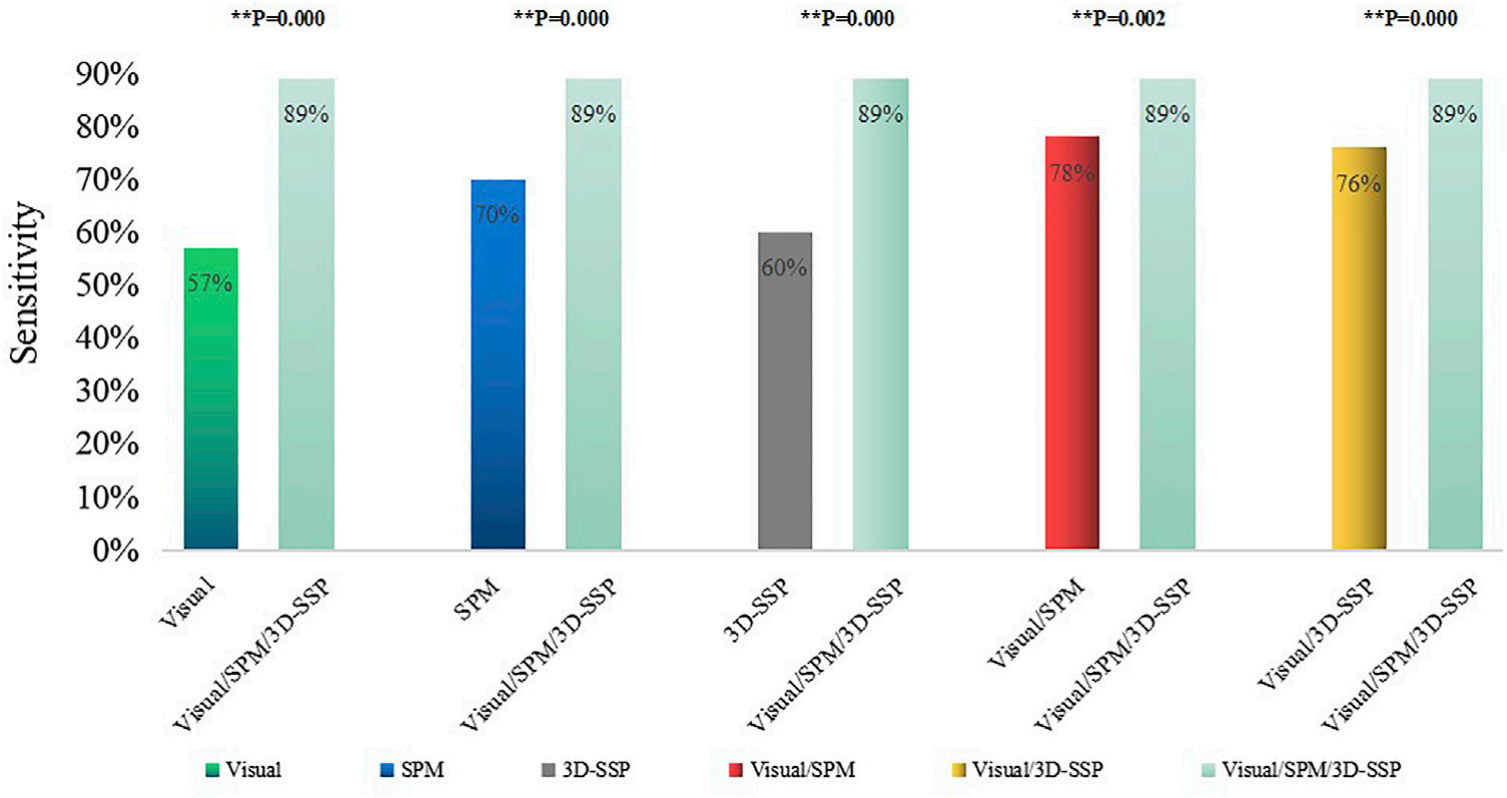
Comparison of the sensitivities of single method, two combined methods, three combined methods (**p < 0.05, ##*p* > 0.05). Visual/SPM/3D-SSP, Visual assessment combined SPM and 3D-SSP.

The sensitivity of three methods combined was significantly higher than any single method or two-method combination, and the comparison between them was statistically significant (Figure 4).

## Discussion

In previous studies, when judging the ability of different methods to detect epileptic foci, localization of epileptic foci was based on multidisciplinary comprehensive evaluation or SEEG localization results (Gonzalez-Martinez et al., 2013). However, both approaches are susceptible to subjective errors and are not sufficient for the most accurate location of epileptic foci. We used the regions of surgical resection of patients with over 1 year follow-up and no seizures as standard epileptic foci, allowing us to assess the true efficacies of different methods. Due to the immature brain development and the boundary between white and gray matter is unclear of people under 5 years old, so we did not include people under 5 years old in this study.

Previous researches have shown that, although ^18^F-FDG PET is highly sensitive in detecting epileptic foci, the abnormal metabolic regions detected by ^18^F-FDG PET are often larger than the real epileptic foci (Rathore et al., 2014; Uijl et al., 2007). The main function of ^18^F-FDG PET is to detect potential epileptic foci, after which epileptic foci can be comprehensively located in combination with other examinations. Previous studies have shown that the sensitivity of visual assessment based on ^18^F-FDG PET in detecting epileptic foci in patients with refractory epilepsy ranges from 36 to 78.2% (Spencer, 1994; Kim et al., 2002; Desai et al., 2013; Wang et al., 2016; Jayalakshmi et al., 2019; Rossi Sebastiano et al., 2020), while the sensitivity of visual assessment based on ^18^F-FDG PET to detect epileptic foci in patients with MRI-negative refractory epilepsy ranges from 36 to 75% (Kim et al., 2002; Rossi Sebastiano et al., 2020). Our study showed that visual assessment correctly detected 52 out of 91 patients, with a sensitivity of 57%. Although a previous study found that the sensitivity of visual assessment based on ^18^F-FDG PET was 75% in MRI-negative refractory epilepsy, SEEG results were used as the reference criteria for localization of epileptic foci (Rossi Sebastiano et al., 2020). Another study found that the sensitivity of visual assessment based on ^18^F-FDG PET in MRI-negative frontal lobe patients was only 36%, although postoperative results were used as the standard for localization of epileptic foci (Kim et al., 2002). These differences are due to the complex functional changes observed in patients with frontal lobe epilepsy, which can lead to inaccurate localization of ^18^F-FDG PET. Our study included patients with MRI-negative epilepsy and postoperative compliance with Engel’s Class I criteria, and our results demonstrate the true clinical value of visual assessment based on ^18^F-FDG PET in MRI-negative refractory epilepsy. Although our study was based on postoperative follow-up results, the sensitivity of visual assessment in our study was within the range of previous results.

Previous studies have shown that the sensitivity of SPM in detecting epileptic foci in patients with refractory epilepsy ranges from 40 to 83% (Archambaud et al., 2013; Kim et al., 2002; Kumar et al., 2010; Mayoral et al., 2016; Wang et al., 2016). No papers about the sensitivity of SPM in detecting epileptic foci in patients with MRI-negative refractory epilepsy have been previously published. Most studies have reported that the sensitivity of SPM is higher than that of visual assessment. There have been two studies reporting the sensitivity of SPM as 83 and 79%, respectively (Archambaud et al., 2013; Wang et al., 2016). However, the definition of epileptic foci was based on the results of preoperative assessment in these two studies. Kumar et al. showed that the sensitivity of SPM is 71% in detecting epileptic foci in patients with refractory epilepsy based on a lack of seizures after surgery (Kumar et al., 2010). In our study, the sensitivity of SPM in MRI-negative epilepsy patients was 70% based on lack of post-surgery seizures, and our results were similar to those of Kumar et al. (2010). Although most of the previous results reported a high sensitivity of SPM in detecting refractory epileptic foci, one study, which included patients that were ^18^F-FDG PET-negative, determined that the sensitivity of SPM was only 40% (Mayoral et al., 2016). SPM results were based on ^18^F-FDG PET images, so a study based on ^18^F-FDG PET-negative patients will suggest reduced sensitivity of SPM.

**FIGURE 5:**
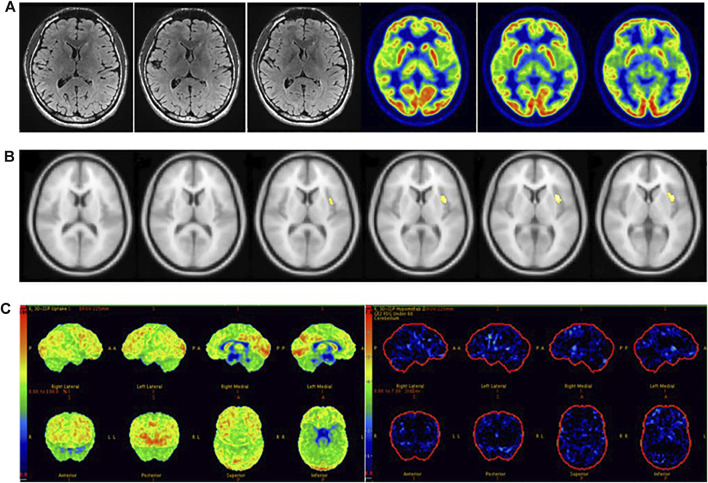
Male, 22 years old, left insula epilepsy. **(A)** No structural lesions causing seizures were found at MRI 3.0 T epilepsy-protocol. ^18^F-FDG PET showed doubtful abnormal decreased metabolism in the left insula lobe. **(B)** SPM did not find abnormal metabolic regions whlie *p* < 0.005 and K > 200 but obtain abnormal metabolism in left insula while *p* < 0.005 and K > 50. **(C)** 3D-SSP did not obtain abnormal metabolic regions. The left insula was surgically resected and lack of seizures for 2 years follow-up.

The sensitivity of SPM in detecting epileptic foci is not only related to the localization standard of epileptic foci, but also to the setting results of threshold (P) and voxel (K), for which there are still no unified setting standards. High *p*-value matching big voxel and low *p*-value matching small voxel were the most common threshold settings in previous studies. In our analysis of previous studies (Archambaud et al., 2013; Kim et al., 2002; Kumar et al., 2010; Mayoral et al., 2016; Wang et al., 2016), we found that *p* < 0.005 was most sensitive when both children and adults were included in the study, while *p* < 0.001 was more sensitive when only children were included in the study. The patients in our study included children and adults, and we concluded that the sensitivity of SPM in detecting epileptic foci was highest when *p* < 0.005 (uncorrected), in agreement with two previous studies (Kim et al., 2002; Mayoral et al., 2016). We established an optimized scheme for SPM. First, ^18^F-FDG PET images of all patients with MRI-negative refractory epilepsy were analyzed with *p* < 0.005, K > 200 and obtained abnormal metabolic regions were defined as potential epileptic foci. For patients without abnormal metabolic regions, further analysis was performed with *p* < 0.005, K > 100. For patients who still exhibited no abnormal metabolic zones after the previous threshold analysis, we continued the analysis with *p* < 0.005, K > 50. By keeping the *p*-value unchanged and gradually reducing the K value, we were able to identify more areas with minor metabolic abnormalities, which could be easily ignored with larger K values or using visual evaluation (Figure 5). We found that correction would reduce the sensitivity of SPM. Preoperative localization of epileptic foci is the result of multidisciplinary evaluation rather than a single examination. The high sensitivity of ^18^F-FDG PET ensures that potential epileptic foci leading to seizures will not be missed, while SPM correction will limit this function to some extent. We conclude that SPM could play a beneficial role in the detection of epileptic foci when appropriate thresholds and voxels are chosen without correction.

3D-SSP has been used previously to detect abnormal metabolism in the brains of patients with early Alzheimer’s disease (AD) (Imabayashi et al., 2004; Kaneko et al., 2004; Kirino, 2017; Lehman et al., 2012; Minoshima et al., 2020; Tang et al., 2004). Since the Cortex ID database contains ^18^F-FDG PET images of normal subjects, a 3D-SSP method based on the Cortex ID database has recently been used to detect epileptic foci in patients with refractory epilepsy. Our study showed that the sensitivity of 3D-SSP to detect epileptic foci was 60%. So far, only one paper has applied 3D-SSP to the localization of epileptic foci (Wang et al., 2016). The study concluded that the sensitivity of 3D-SSP in detecting epileptic foci (71%) was higher than that reported in our study (60%). In their study, the localization of epileptic foci was based on preoperative comprehensive evaluation, while all the patients in our study met Engel’s Class Ⅰ criteria after surgery. Additionally, all patients in our study were MRI-negative. These differences contributed to the differences in results between the two studies. Previous studies have suggested that Z-values greater than 1.96 or 1.5 for 3D-SSP analysis based on SPECT images were useful for diagnosing AD patients (Kaneko et al., 2004; Wang et al., 2016; Kirino, 2017). However, the optimal Z threshold for 3D-SSP analysis based on ^18^F-FDG PET image for the detection of epileptic foci in patients with refractory epilepsy has not yet been determined. The previous study did not report a specific Z-value (Wang et al., 2016). In our study, thresholds were set according to confidence intervals, and we concluded not only that the sensitivity of 3D-SSP was high, but also that the number of abnormal metabolic regions aligned with epileptic foci was similar to visual assessment at a Z-value of 1.28.

Visual assessment, SPM and 3D-SSP based on ^18^F-FDG PET had high sensitivity in detecting epileptic foci, but their ability to correctly locate epileptic foci was low. This was due to the low specificity of ^18^F-FDG PET (Rathore et al., 2014; Uijl et al., 2007), meaning that the abnormal metabolic regions detected by SPM and 3D-SSP based on ^18^F-FDG PET were often larger than the real epileptic foci. Previous articles did not indicate the specificities of SPM and 3D-SSP in locating epileptic foci (Archambaud et al., 2013; Kim et al., 2002; Kumar et al., 2010; Mayoral et al., 2016; Wang et al., 2016). In our study, the specificities of SPM or 3D-SSP analysis based on ^18^F-FDG PET were not significantly improved.

**FIGURE 6:**
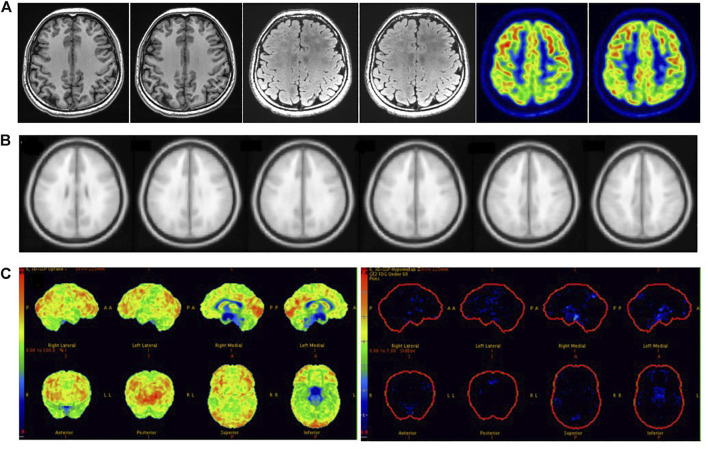
Female, 21 years old, right frontal lobe epilepsy. **(A)** No structural lesions causing seizures were found at MRI 3.0 T epilepsy-protocol. ^18^F-FDG PET obtained abnormal decreased metabolism in the left frontal, parietal and temporal lobes. **(B)** SPM obtained abnormal metabolism in left temporal lobe while *p* < 0.005 and K > 100. **(C)** 3D-SSP obtained abnormal metabolism in right frontal, double parietal and double occipital lobes. Further SEEG examination obtained the epilepsy foci originated from right frontal lobe. There had been no seizures for 3 years after surgical resection.

In clinical work, visual assessment based on ^18^F-FDG PET images has many uncertain factors, and SPM and 3D-SSP can reduce human factors to allow the objective detection of epileptic foci. We found that the sensitivity of SPM was significantly higher than that of visual assessment, and there was a statistically significant difference between the two methods. SPM correctly detected 19 of the 39 patients incorrectly detected by visual assessment, with epileptic foci mainly located in small areas with low resolution that could be easily overlooked by visual analysis. We found that patients with abnormal metabolic regions located in the insula were more often identified by SPM than in visual assessment; because the insula is small in size and located at the junction of several brain lobes, it is difficult to assess visually (Figure 5). Ajay Kumar et al. also concluded that SPM is more useful for in cases of medial epilepsy, because medial abnormal metabolic foci are more likely to be missed by visual assessment (Kumar et al., 2010). Although there was no statistically significant difference between 3D-SSP and visual assessment, 3D-SSP was able to detect an additional 17 of the 39 patients who were incorrectly detected by visual assessment. 3D-SSP was not as good as SPM at detecting medial epileptic foci, but it was still able to detect microscopic abnormal metabolic areas of the cortex. In 10 patients, we found that neither visual assessment nor SPM correctly detected epileptic foci, but 3D-SSP correctly detected the foci (Figure 6). Although there was no statistically significant difference between the sensitivities of the two tests, 3D-SSP detected fewer epileptic foci than SPM, mainly because it failed to correctly detect epileptic foci in 14 of the 52 patients who were correctly detected by visual assessment. 3D-SSP software can be used to diagnose degenerative diseases in the elderly by analyzing metabolic changes of ^18^F-FDG PET. The age of control group in 3D-SSP database is >40 years old, and the age of the control group we selected (40–60 years old) is older than the average age of epilepsy group (21 years old), so there will be some deviation in the results obtained. If the 3D-SSP software database adds a young control population, it will provide more help for patients with refractory epilepsy.

While Visual/3D-SSP did not significantly improve sensitivity compared with SPM, visual assessment combined with either method did significantly improve sensitivity compared with visual assessment, SPM or 3D-SSP alone. SPM and 3D-SSP can objectively detect microscopic lesions overlooked in visual assessment, while visual assessment can identify lesions below the threshold settings of SPM or 3D-SSP. Therefore, the combination of the two methods can significantly improve sensitivity. Wang et al. also concluded that the combined sensitivity of the two tests is higher than that of the single test (Wang et al., 2016). There was no statistically significant difference between the sensitivities of Visual/SPM and Visual/3D-SSP, suggesting that similar sensitivity can be achieved in clinical application by combining visual assessment with either of the two objective methods.

**FIGURE 7:**
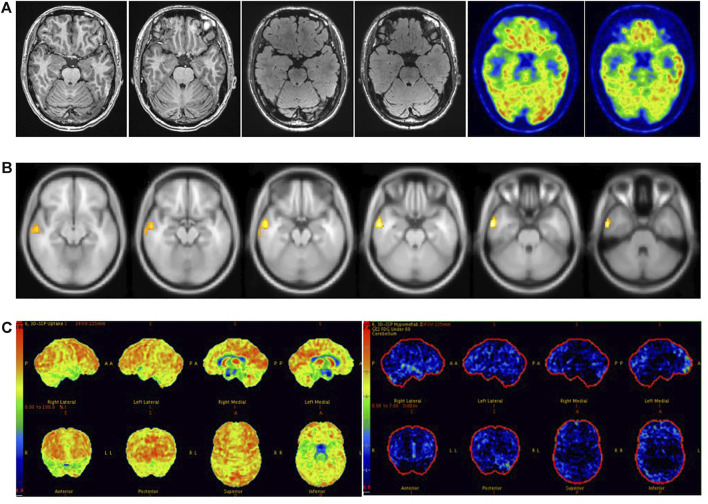
Male, 37 years old, right temporal lobe epilepsy. **(A)** No structural lesions causing seizures were found at 3.0 T epilepsy-protocol. ^18^F-FDG PET obtained abnormal decreased metabolism in the right temporal lobe. **(B)** SPM showed decreased metabolism in right temporal lobe while *p* < 0.005 and K > 200. **(C)** Abnormal metabolism in the right temporal lobe was found by 3D-SSP. The right anterior temporal lobe was surgically resected and remained seizure-free for 2 years follow-up.

Sensitivity reached 89% when visual assessment was combined with SPM and 3D-SSP, higher than for either method. A study has reported that the sensitivity of ^18^F-FDG PET combined with three other methods to detect epileptic foci in patients with MRI-negative refractory epilepsy can reach 80% (Rossi Sebastiano et al., 2020), and another paper concluded that the sensitivity of Visual/SPM/3D-SSP can reach 97% (Wang et al., 2016). Our study is the first to simultaneously combine visual assessment, SPM and 3D-SSP based on ^18^F-FDG PET to detect epileptic foci in MRI-negative epilepsy patients. We found that the combination of the three methods can significantly improve sensitivity, but reduces consistency due to the complementary detection capacities of the three methods, resulting in a larger range of detected abnormal lesions than actual epileptic foci. Therefore, in clinical work, we choose methods according to different situations. When the visual assessment is consistent with the electroclinical information of patients with MRI-negative refractory epilepsy, additional objective testing methods may not be accepted. When visual assessment is inconsistent with the patient’s electroclinical information, we need to further accept objective testing methods and even combine visual assessment with multiple objective testing methods to prevent missing epileptogenic foci. When receiving multiple methods, judgments must be made based on different results. Attention should be paid to cases in which different methods indicate the same lesion, as it is the most likely potential epileptic focus (Figure 7). If multiple methods are inconsistent and cannot be used for localization, however, further evaluation should be performed by combining clinical symptoms with video-EEG or even SEEG (Figure 6).

SPM and 3D-SSP can be used as objective methods to detect epileptic foci before surgery in patients with MRI-negative refractory epilepsy. Clinical routine visual assessment of PET images is preferred in patients with MRI-negative refractory epilepsy. When visual assessment is consistent with electroclinical information of patients with epilepsy, additional objective testing methods are not acceptable. When the visual assessment is inconsistent with the patient’s electroclinical information, SPM or 3D-SSP was further selected to assess the epileptic foci. If the combination of the two methods still fails to accurately locate the epileptic foci, comprehensive evaluation can be performed by combining the three methods.

## Data Availability

The raw data supporting the conclusions of this article will be made available by the authors, without undue reservation.

## References

[B1] Archambaud F. Bouilleret V. Hertz-Pannier L. Chaumet-Riffaud P. Rodrigo S. Dulac O. (2013). Optimizing Statistical Parametric Mapping Analysis of 18F-FDG PET in Children. EJNMMI Res. 3, 2. 10.1186/2191-219X-3-2 23289862 PMC3558387

[B2] Bernasconi A. Cendes F. Theodore W. H. Gill R. S. Koepp M. J. Hogan R. E. (2019). Recommendations for the Use of Structural Magnetic Resonance Imaging in the Care of Patients with Epilepsy: A Consensus Report from the International League against Epilepsy Neuroimaging Task Force. Epilepsia 60, 1054–1068. 10.1111/epi.15612 31135062

[B3] Chassoux F. Rodrigo S. Semah F. Beuvon F. Landre E. Devaux B. (2010). FDG-PET Improves Surgical Outcome in Negative MRI Taylor-type Focal Cortical Dysplasias. Neurology 75, 2168–2175. 10.1212/WNL.0b013e31820203a9 21172840

[B4] Desai A. Bekelis K. Thadani V. M. Roberts D. W. Jobst B. C. Duhaime A.-C. (2013). Interictal PET and Ictal Subtraction SPECT: Sensitivity in the Detection of Seizure Foci in Patients with Medically Intractable Epilepsy. Epilepsia 54, 341–350. 10.1111/j.1528-1167.2012.03686.x 23030361

[B5] Duncan J. S. Winston G. P. Koepp M. J. Ourselin S. (2016). Brain Imaging in the Assessment for Epilepsy Surgery. Lancet Neurol. 15, 420–433. 10.1016/s1474-4422(15)00383-x 26925532 PMC6736670

[B6] Dwivedi R. Ramanujam B. Chandra P. S. Sapra S. Gulati S. Kalaivani M. (2017). Surgery for Drug-Resistant Epilepsy in Children. N. Engl. J. Med. 377, 1639–1647. 10.1056/NEJMoa1615335 29069568

[B7] Engel J. Jr. McDermott M. P. Wiebe S. Langfitt J. T. Stern J. M. Dewar S. (2012). Early Surgical Therapy for Drug-Resistant Temporal Lobe Epilepsy. JAMA 307, 922–930. 10.1001/jama.2012.220 22396514 PMC4821633

[B8] Gonzalez-Martinez J. Bulacio J. Alexopoulos A. Jehi L. Bingaman W. Najm I. (2013). Stereoelectroencephalography in the "difficult to Localize" Refractory Focal Epilepsy: Early Experience from a North American Epilepsy center. Epilepsia 54, 323–330. 10.1111/j.1528-1167.2012.03672.x 23016576

[B9] Hwang S. I. Kim J. H. Park S. W. Han M. H. Yu I. K. Lee S. H. (2001). Comparative Analysis of MR Imaging, Positron Emission Tomography, and Ictal Single-Photon Emission CT in Patients with Neocortical Epilepsy. AJNR Am. J. Neuroradiol 22, 937–946. 10.1016/S0925-4927(01)00075-0 11337340 PMC8174931

[B10] Imabayashi E. Matsuda H. Asada T. Ohnishi T. Sakamoto S. Nakano S. (2004). Superiority of 3-dimensional Stereotactic Surface Projection Analysis over Visual Inspection in Discrimination of Patients with Very Early Alzheimer's Disease from Controls Using Brain Perfusion SPECT. J. Nucl. Med. 45, 1450–1457. 15347711

[B11] Jayalakshmi S. Nanda S. K. Vooturi S. Vadapalli R. Sudhakar P. Madigubba S. (2019). Focal Cortical Dysplasia and Refractory Epilepsy: Role of Multimodality Imaging and Outcome of Surgery. AJNR Am. J. Neuroradiol 40, 892–898. 10.3174/ajnr.A6041 31000525 PMC7053892

[B12] Jones A. L. Cascino G. D. (2016). Evidence on Use of Neuroimaging for Surgical Treatment of Temporal Lobe Epilepsy. JAMA Neurol. 73, 464–470. 10.1001/jamaneurol.2015.4996 26926529

[B13] Kaneko K. Kuwabara Y. Sasaki M. Ogomori K. Ichimiya A. Koga H. (2004). Posterior Cingulate Hypoperfusion in Alzheimerʼs Disease, Senile Dementia of Alzheimer Type, and Other Dementias Evaluated by Three-Dimensional Stereotactic Surface Projections Using Tc-99m HMPAO SPECT. Clin. Nucl. Med. 29, 362–366. 10.1097/01.rlu.0000127091.43180.92 15166883

[B14] Kim Y. K. Lee D. S. Lee S. K. Chung C. K. Chung J. K. Lee M. C. (2002). (18)F-FDG PET in Localization of Frontal Lobe Epilepsy: Comparison of Visual and SPM analysisF-FDG PET in Localization of Frontal Lobe Epilepsy: Comparison of Visual and SPM Analysis. J. Nucl. Med. 43, 1167–1174. 10.1590/S1517-86922003000400006 12215554

[B15] Kirino E. (2017). Three-dimensional Stereotactic Surface Projection in the Statistical Analysis of Single Photon Emission Computed Tomography Data for Distinguishing between Alzheimer's Disease and Depression. Wjp 7, 121–127. 10.5498/wjp.v7.i2.121 28713690 PMC5491477

[B16] Kumar A. Juhász C. Asano E. Sood S. Muzik O. Chugani H. T. (2010). Objective Detection of Epileptic Foci by 18F-FDG PET in Children Undergoing Epilepsy Surgery. J. Nucl. Med. 51, 1901–1907. 10.2967/jnumed.110.075390 21078805 PMC3157889

[B17] Kwan P. Arzimanoglou A. Berg A. T. Brodie M. J. Allen Hauser W. Mathern G. (2010). Definition of Drug Resistant Epilepsy: Consensus Proposal by the Ad Hoc Task Force of the ILAE Commission on Therapeutic Strategies. Epilepsia 51, 1069–1077. 10.1111/j.1528-1167.2009.02397.x 19889013

[B18] Lehman V. T. Carter R. E. Claassen D. O. Murphy R. C. Lowe V. Petersen R. C. (2012). Visual Assessment versus Quantitative Three-Dimensional Stereotactic Surface Projection Fluorodeoxyglucose Positron Emission Tomography for Detection of Mild Cognitive Impairment and Alzheimer Disease. Clin. Nucl. Med. 37, 721–726. 10.1097/RLU.0b013e3182478d89 22785496

[B19] Mayoral M. Marti-Fuster B. Carreño M. Carrasco J. L. Bargalló N. Donaire A. (2016). Seizure-onset Zone Localization by Statistical Parametric Mapping in Visually normal18F-FDG PET Studies. Epilepsia 57, 1236–1244. 10.1111/epi.13427 27286896

[B20] Minoshima S. Frey K. A. Koeppe R. A. Foster N. L. Kuhl D. E. (2020). A Diagnostic Approach in Alzheimer's Disease Using Three-Dimensional Stereotactic Surface Projections of Fluorine-18-FDG PET. J. Nucl. Med. 61, 142s–152s. 10.2967/jnumed.120.252510a 7790950

[B21] Mizumura S. Kumita S.-i. (2006). Stereotactic Statistical Imaging Analysis of the Brain Using the Easy Z-Score Imaging System for Sharing a normal Database. Radiat. Med. 24, 545–552. 10.1007/s11604-006-0056-8 17058152

[B22] Muhlhofer W. Tan Y. L. Mueller S. G. Knowlton R. (2017). MRI ‐negative Temporal Lobe Epilepsy-What Do We Know? Epilepsia 58, 727–742. 10.1111/epi.13699 28266710

[B23] Rathore C. Dickson J. C. Teotónio R. Ell P. Duncan J. S. (2014). The Utility of 18F-Fluorodeoxyglucose PET (FDG PET) in Epilepsy Surgery. Epilepsy Res. 108, 1306–1314. 10.1016/j.eplepsyres.2014.06.012 25043753

[B24] Rossi Sebastiano D. Tassi L. Duran D. Visani E. Gozzo F. Cardinale F. (2020). Identifying the Epileptogenic Zone by Four Non-invasive Imaging Techniques versus Stereo-EEG in MRI-Negative Pre-surgery Epilepsy Patients. Clin. Neurophysiol. 131, 1815–1823. 10.1016/j.clinph.2020.05.015 32544836

[B25] Spencer S. S. (1994). The Relative Contributions of MRI, SPECT, and PET Imaging in Epilepsy. Epilepsia 35 (Suppl. 6), S72–S89. 10.1111/j.1528-1157.1994.tb05990.x 8206016

[B26] Tang B.-N. -T. Minoshima S. George J. Robert A. Swine C. Laloux P. (2004). Diagnosis of Suspected Alzheimers? Disease Is Improved by Automated Analysis of Regional Cerebral Blood Flow. Eur. J. Nucl. Med. Mol. Imaging 31, 1487–1494. 10.1007/s00259-004-1597-7 15232656

[B27] Uijl S. G. Leijten F. S. S. Arends J. B. A. M. Parra J. van Huffelen A. C. Moons K. G. M. (2007). The Added Value of [18F]-Fluoro-D-Deoxyglucose Positron Emission Tomography in Screening for Temporal Lobe Epilepsy Surgery. Epilepsia 48, 2121–2129. 10.1111/j.1528-1167.2007.01197.x 17651417

[B28] von Oertzen J. Urbach H. Jungbluth S. Kurthen M. Reuber M. Fernandez G. (2002). Standard Magnetic Resonance Imaging Is Inadequate for Patients with Refractory Focal Epilepsy. J. Neurol. Neurosurg. Psychiatry 73, 643–647. 10.1136/jnnp.73.6.643 12438463 PMC1757366

[B29] Wang K. Liu T. Zhao X. Xia X. Zhang K. Qiao H. (2016). Comparative Study of Voxel-Based Epileptic Foci Localization Accuracy between Statistical Parametric Mapping and Three-Dimensional Stereotactic Surface Projection. Front. Neurol. 7, 164. 10.3389/fneur.2016.00164 27729898 PMC5037321

[B30] Wieser H. G. Blume W. T. Fish D. Goldensohn E. Hufnagel A. King D. (2001). ILAE Commission Report. Proposal for a New Classification of Outcome with Respect to Epileptic Seizures Following Epilepsy Surgery. Epilepsia 42, 282–286. 10.1046/j.1528-1157.2001.35100.x 11240604

[B31] World Health Organization (2019). Epilepsy. Available at: https://www.who.int/zh/news-room/fact-sheets/detail/epilepsy (Accessed June 20, 2019).

